# RNA Binding by the m6A Methyltransferases METTL16 and METTL3

**DOI:** 10.3390/biology13060391

**Published:** 2024-05-29

**Authors:** Kyle D. Mansfield

**Affiliations:** Biochemistry and Molecular Biology Department, Brody School of Medicine, East Carolina University, Greenville, NC 27834, USA; mansfieldk@ecu.edu

**Keywords:** METTL3, METTL16, RNA binding, N^6^-methyladenosine, m6A

## Abstract

**Simple Summary:**

Interest in RNA methylation has surged in recent years with the identification of the major eukaryotic mRNA m6A methyltransferases, METTL3 and METTL16. Interestingly, both METTL3 and METTL16 have “methylation-independent” functions that have been discovered, including translational regulation. In this review, we will introduce RNA m6A methylation and discuss the varying roles that these enzymes perform, including their possible roles as methylation-independent RNA binding proteins.

**Abstract:**

Methyltransferases are a wide-ranging, yet well-conserved, class of molecules that have been found to modify a wide variety of substrates. Interest in RNA methylation has surged in recent years with the identification of the major eukaryotic mRNA m6A methyltransferase METTL3. METTL16 has also been identified as an RNA m6A methyltransferase; however, much less is known about its targets and actions. Interestingly, in addition to their catalytic activities, both METTL3 and METTL16 also have “methylation-independent” functions, including translational regulation, which have been discovered. However, evidence suggests that METTL16’s role as an RNA-binding protein may be more significant than is currently recognized. In this review, we will introduce RNA methylation, specifically m6A, and the enzymes responsible for its deposition. We will discuss the varying roles that these enzymes perform and delve deeper into their RNA targets and possible roles as methylation-independent RNA binding proteins. Finally, we will touch upon the many open questions still remaining.

## 1. N^6^-Methyladenosine Modification of RNA

Encoded within our DNA are the instructions to produce all of the proteins required for our cells to function properly. In the process of going from DNA to protein, there are a number of regulatory steps at the transcriptional, post-transcriptional, and translational levels. Interest in post-transcriptional and translational regulation has increased in recent years, in part due to the regulatory potential that has been discovered. One such discovery that has generated a lot of interest is the N^6^-methyladenosine (m6A) modification of messenger ribonucleic acid (mRNA). m6A is just one of over 100 RNA modifications that can be found in eukaryotic cells [1]. Modifications of ribosomal RNA (rRNA) and transfer RNA (tRNA) have been studied for quite some time and are known to regulate various functions of these RNAs. For example, pseudouridine has well-established roles in both rRNA and tRNA, and studies are now revealing emerging functions in mRNA [2,3,4,5]. Many of the rRNA and tRNA modifications studied to date are involved in fine-tuning translational efficiency, ultimately regulating protein output [6]. While there remain many unknowns about mRNA modifications, at least 13 modifications, including m6A, 5-methylcytidine (m5C), and 7-methylguanosine (m7G), have been found, although the function of many has yet to be discovered [1].

Much more is known about m6A than the other mRNA modifications. It has been estimated that there are approximately three m6As per mRNA, making it the most abundant modification found in mRNA to date [7,8]. m6A has been reported to affect all aspects of mRNA regulation, from splicing and processing of the pre-mRNA to the stability and translational efficiency of the mature mRNA [9,10,11,12,13,14]. Biologically, m6A has been shown to be involved in almost every aspect of cellular function, as well as in numerous processes including cancer progression and stem cell development [15,16,17,18,19]. As with other posttranscriptional regulatory mechanisms, m6A has also been shown to be involved in rapid adaptation to changing cellular conditions. For example, we and others have found m6A to be involved in the hypoxic response [14,20], as well as in other cellular stress responses [21,22,23].

m6A is deposited on RNA by a family of RNA m6A methyltransferases that includes Methyltransferase-like 3 (METTL3), METTL16, METTL5 and ZCCHC4. In-depth insight into these m6A “writers” can be found in a number of excellent recent reviews [24,25,26,27,28,29,30,31]. METTL5 and ZCCHC4 have been shown to methylate 18S and 28S rRNA, respectively, and likely contribute to translational fidelity [32,33,34]. The major mRNA methyltransferase complex in eukaryotic cells consists of METTL3 and METTL14, the Wilms’ tumor-associating protein (WTAP), and other accessory proteins such as KIAA1429 and RBM15. The major role of this complex is to methylate nascent pre-mRNA within the nucleus [35,36,37,38,39,40,41]. METTL3 is the catalytic subunit of the complex and utilizes S-adenosyl methionine (SAM) as a substrate to methylate target adenosines on the nitrogen at the 6th carbon residue, contained within a DRACH m6A consensus sequence (D = A, T, or G (adenine, thymine or guanine), R = A or G (purine), where A is the methylated base, C = cytosine, and H = A, C, or T). This motif is most often found in 3′ UTRs and around both start and stop codons [35,42,43,44,45]. METTL14 lacks catalytic activity but participates in mRNA binding/targeting [46,47,48]. The third member of the complex, WTAP, is not required for methylation; however, its presence enhances methylation activity and is responsible for the localization of the complex to the nuclear speckle [36,49]. Interestingly, although the complex is found predominantly in the nucleus, where it is thought to methylate pre-mRNA, the methyltransferase complex, along with methyltransferase activity, has also been found within the cytoplasm [36,50,51,52,53]. This raises the possibility of additional functions in regulating mature mRNA, some of which are discussed later.

The RNA m6A methyltransferase METTL16 was originally classified as an rRNA methyltransferase, due to its homology with the *E. coli* m6A methyltransferase, YbiN. However, numerous reports now show it binding to multiple classes of RNA, including mRNA, rRNA, lncRNA, and snRNA [54,55,56,57,58]. Interestingly, to date, only two targets (MAT2A mRNA and U6 snRNA) have been verified to be directly methylated by METTL16 [56]. Like other RNA methyltransferases, METTL16 contains the Rossman-like fold of class I methyltransferases and uses SAM as the methyl donor. The two verified targets identified share the RNA methylation consensus sequence of UACAGAGAA (the second A (underlined), found between the C and G, is the methylated base) and, unlike METTL3, METTL16 appears to also require a specific secondary structure to properly methylate its RNA targets [24]. In addition to the methyltransferase domain, METTL16 protein has two RNA-binding domains, one of which resides in an arginine-rich vertebrate-conserved region (VCR). METTL16 has many reported roles in the cell and evidence suggests that it is essential for most cells [59,60,61,62]. It has also been found to be located in the cytoplasm as well as in the nucleus [55,57,58,63].

An intriguing aspect of m6A is that the modification is reversible and can be removed by the demethylase alkylation repair homolog 5 (ALKBH5) and fat mass and obesity-related protein (FTO) [64,65,66,67,68,69,70,71,72,73]. These enzymes are part of the ALKB family, which are historically regarded as Fe^II^ and α-ketoglutarate-dependent nucleic acid repair enzymes [74,75]. Interestingly, while ALKBH5 and FTO belong to the same family of enzymes, their mechanisms of m6A demethylation differ. The FTO demethylation of m6A results in two intermediate nucleotides, N^6^-hydroxymethyladenosine (hm6A) and N^6^-formyladenosine (fm6A), while ALKBH5 demethylation produces no detectable intermediate nucleotides [69,76]. In addition, FTO has been shown to preferentially demethylate N^6^,2′-*O*-dimethyladenosine (m6Am), suggesting that the major cellular m6A demethylase is ALKBH5 [77]. Further insight into m6A demethylation by FTO and ALKBH5 can be found in recent reviews [78,79]. 

Modification of mRNA by m6A serves to alter the mRNA’s fate. However, it does this by acting as a dynamic binding site for a number of RNA-binding proteins (RBPs) that preferentially recognize the modified mRNA [80,81,82]. There are RBPs that bind specifically to the methylated mRNA, including many members of the YTH domain family [12,83,84], as well as the insulin-like growth factor 2 mRNA-binding protein (IGF2BP) family [85]. m6A has also been shown to affect the binding of RBPs that do not directly recognize the m6A, but instead may be affected by other aspects of the methylation, including changes in RNA secondary structure [86,87]. 

While the broader consequences of mRNA methylation are still being investigated, we do know some of the effects of mRNA methylation that are determined by the m6A binding proteins that ultimately recognize the modified mRNA. Initial studies suggested that m6A modification marked the mRNA for degradation via YTHDF2 recognition and the degradation of the mRNA through DCP1/DCP2 decapping and CCR4-NOT deadenylation [9,12,41,65,81]. Further studies into the YTH family of RNA-binding proteins suggested that each regulated the fate of m6A methylated mRNA differently. YTHDF1 was reported to stimulate translational efficiency by interacting with initiation factors to enhance the ribosomal loading of the methylated mRNA, while YTHDF3 and YTHDC2 were reported to promote the translation of m6A methylated mRNA through other mechanisms [11,13,88]. YTHDC1 on the other hand, has been shown to alter splicing through the recruitment of splicing factors to the modified mRNA [84,89]. In contrast to the YTH family, the IGF2BP family has been shown to stabilize and facilitate the increased translation of m6A methylated mRNAs [85,90]. Thus, it does appear that, depending on the m6A reader that recognizes the mRNA, modification can have a multitude of effects. However, more recent studies suggest that mRNA degradation is the major effect of m6A modification, especially with regard to recognition by the TYHDF reader proteins, which appear to be redundant in function [91]. Whether this is true of other reader proteins and/or protein families will require more careful analysis of the data.

## 2. RNA Binding by the mRNA Methyltransferases METTL3 and METTL16

**Figure 1:**
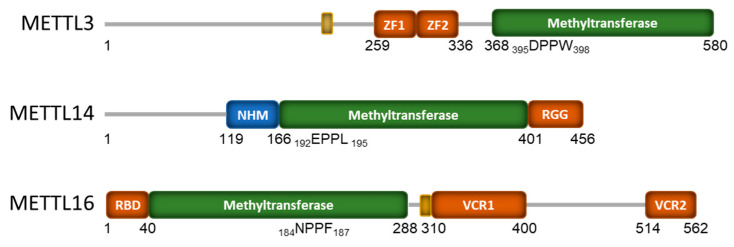
Domain structure of the human mRNA m6A methyltransferase proteins METTL3, METTL14, and METTL16. The numbers indicate amino acids from the N to C terminals, with methyltransferase domain shown in green and the catalytic site amino acids indicated. RNA-binding domains are shown in orange, while the small yellow boxes indicate nuclear localization signals (NLS). The zinc finger (ZF), N-terminal α-helical motif (NHM) is shown in blue; the arginine-rich region (RGG), N-terminal RNA-binding domain (RBD), and vertebrate-conserved regions (VCR) are also indicated.

While much focus has been paid to the methyltransferase activity of both METTL3/14 and METTL16, they have both been described as also having methyltransferase-independent functions. These functions require RNA binding and reportedly affect translational activity, albeit by different mechanisms. This may be because METTL16 and METTL3/14 have very different domain architectures (Figure 1) and use vastly different mechanisms to bind to their RNA targets [26].

### 2.1. METTL3/METTL14 RNA Binding

Separately, METTL3 and METTL14 exhibit very weak in vitro methyltransferase activity and need to be in complex with one another for full catalytic activity. Interestingly, catalytic activity requires full-length METTL3, while only the central methyltransferase domain of METTL14 is required [46]. METTL3 consists of an N-terminal extension that contains a nuclear localization signal (NLS). This is followed by a zinc finger domain that contains two consecutive CCCH zinc finger motifs (ZF1 and ZF2), a disordered linker, and a C-terminal methyltransferase domain (Figure 1) with a classic Rossman fold architecture [46,47,48,92]. METTL14 has a non-functional methyltransferase domain which is flanked by an N-terminal helical motif (NHM) and a c-terminal arginine-rich region (RGG; Figure 1). METTL3’s Rossmann fold contains a catalytic loop with the classical DPPW motif, while METTL14 has a nonfunctional EPPL motif (Figure 1). The ZF1 and ZF2 of METTL3 function as RNA recognition domains that help bind the mRNA substrate and orient the DRACH sequence for methylation. However, both subunits of the METTL3/14 complex are required for RNA binding [46]. The C-terminus RGG of METTL14 is positively charged and also contributes to the RNA binding of the complex [93]. METTL3 and METTL14 in complex also form a positively charged groove at their protein:protein interface, which is critical for full methyltransferase activity as it also contributes to interactions with the mRNA substrate [47,48]. More in-depth analysis of the structural aspects of METTL3/14 RNA binding can be found in [26,27,46,48].

### 2.2. METTL16 RNA Binding

In contrast to the METTL3/14 complex, METTL16 functions as an independent monomer [59,94], and a recent publication provides an excellent in-depth review of its structural components [24]. In brief, METTL16 binds its RNA targets through two non-canonical RNA-binding domains. The unique N-terminal RNA-binding domain (RBD) of METTL16 is required for RNA binding and catalysis and is conserved throughout evolution (Figure 1) [59,94,95]. The METTL16 of higher eukaryotes also contains a C-terminal vertebrate-conserved region (VCR) that is required for RNA binding and methylation activity [59,96]. The VCR consists of two arginine-rich domains (VCR1, VCR2) separated by an unstructured linker region (Figure 1). The N-terminal RNA binding domain of METTL16 has a highly conserved, positively charged groove that interacts with the negatively charged phosphate backbone of RNA. This positively charged groove includes multiple lysines and arginines, including two residues (K47 and R279) that have been described to form a claw-like structure that can bind the RNA backbone [95]. Insight into METTL16 binding to the hairpin 1 of MAT2A confirms that METTL16 uses extensive intermolecular contact to clamp the MAT2A hairpin with three long polypeptide segments [94]. In addition to its classic methyltransferase domain with an NPPF catalytic motif, METTL16 has also been found to contain an autoregulatory polypeptide loop (K-Loop) that blocks the SAM binding site and, hence, methylation. Destabilizing the K-Loop, which is found at amino acids 163–167 within the methyltransferase domain, does not affect RNA binding affinity but does activate methyltransferase activity [94]. Many of the early METTL16 structural studies utilized only the N-terminal domain as the unstructured linker region of the C-terminal VCR domain complicated analysis. However, binding experiments utilizing the full-length METTL16 showed a 100-fold stronger affinity for U6 snRNA than the methyltransferase domain alone [96]. This suggests that the C-terminal VCR domain also greatly contributes to RNA binding.

## 3. m6A-Independent Functions of the Methyltransferases

**Figure 2:**
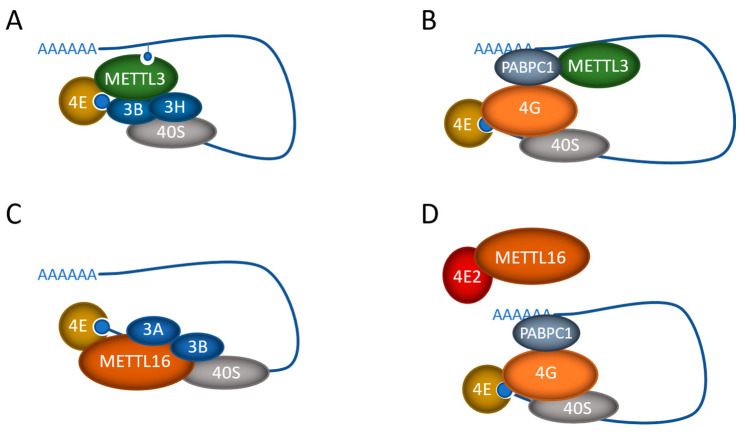
Regulation of translation by the mRNA m6A methyltransferases METTL3 and METTL16. The proposed mechanisms by which METTL3 (**A**,**B**) and METTL16 (**C**,**D**) exert their methylation-independent effects on cancer cell translation. mRNA is shown as the blue line with the polyA tail and cap. Also depicted are the methyltransferase proteins (METTL3, METTL16), polyA binding protein (PABPC1), the 40S ribosome, and eIF4E cap-binding protein, along with eIF4E2, eIF4G, eIF3A, eIF3B, and eIF3H.

While METTL3 and METTL16 both have the catalytic capacity to deposit m6A on the RNA that they bind, both have also been shown to have m6A-independent functions, at least in cancer cells. The effect occurs in the cytoplasm of the cells, in contrast to the canonical nuclear localization of their RNA methylation activity. Interestingly, in both cases, the m6A-independent roles involve the regulation of translation and do not require functional catalytic activity. In most cases, the methyltransferases promote translation initiation through interactions with the initiation machinery (Figure 2).

### 3.1. METTL3 Methylation-Independent Functions

The m6A-independent function of METTL3 was first discovered in lung cancer cells, where METTL3, but not METTL14 or WTAP, was found in the cytoplasm of the cells [53]. Polysome analysis suggested that METTL3 (both wild-type and catalytically inactive) associates with ribosomes and promotes translation in the cytoplasm, and that METTL3 depletion inhibits translation. This effect was attributed to the N-terminus of the METTL3 protein, as it alone was sufficient to promote translation, while the C-terminus, containing the catalytic domain, had no effect on its own. Further work showed that the nuclear cap-binding protein subunit 1/cap-binding protein 80 (NCBP1/CBP80), as well as the eukaryotic translation initiation factor (eIF) eIF4E and the eIF3 subunit eIF3b, co-immunopurified with FLAG-METTL3 in an RNA-independent manner (Figure 2A). However, it is assumed that METTL3 binding to the mRNA was required for the recruitment of the initiation factors, as tethering experiments were able to replicate the increased translation effect. Furthermore, as CBP80 is typically replaced by eIF4E, it is likely not involved in bulk translation but may influence mRNA export or perhaps the pioneer round of translation, which is important for nonsense-mediated decay (NMD) [97,98]. Regardless, METTL3, independent of its methylation activity, was able to promote the translation of specific mRNAs that then supported the increased proliferation and invasion of lung cancer cells [53]. 

In the initial study, it was noted that METTL3 translational targets were enriched in m6A near their stop codons, compared to other m6A-methylated mRNAs [53]. Additional studies confirmed that METTL3 was only able to enhance translation when tethered to reporter mRNA at sites close to the stop codon [99]. This supports a role for METTL3 in RNA looping. In addition, a functional interaction between METTL3 and the eukaryotic translation initiation factor 3 subunit h (eIF3h) was found to be required for METTL3’s association with the other translation initiation factors (Figure 2A). The site of association was narrowed down to a small helical region (150–161 aa) within METTL3 and a single point mutation (A155P) disrupted METTL3’s translational function, while not affecting its interactions with METTL14. Again, these results suggest that METTL3 has two distinct and separable functions within the cell.

A similar effect was also seen in gastric cancer, where, again, METTL3, but not METTL14 or WTAP, was found to be cytoplasmic [100]. Comparison of eCLIP-seq (RNA binding) and meRIP-seq (m6A methylation) data suggested that METTL3 was binding many more mRNAs than it was methylating and that this mRNA binding activity was promoting tumor growth and survival. Interestingly, the overexpression of a catalytic inactive METTL3, or an NLS mutant, had the same functional effect as the overexpression of wild-type protein, again suggesting a cytoplasmic, m6A-independent function. Further investigation revealed that METTL3 promoted translation initiation through its interaction with PABPC1, which stimulated interaction with eIF4F and RNA looping (Figure 2B), similar to the findings of lung cancer studies [100].

While these studies are intriguing, there are a number of questions still remaining. How does METTL3 recognize these mRNAs for translation and is methylation involved in some way (even though it appears to not be necessary), or does it involve other RNA binding factors? Specificity needs to come from somewhere and it is unclear whether it comes directly from METTL3 (perhaps METTL14-independent RNA-binding is different?) or involves other factors. Furthermore, how is METTL3’s cellular localization controlled? What aspects of the cancer cell favor cytoplasmic localization and is it regulatable (and, hence, druggable)? It is also intriguing/confusing that multiple mechanisms have been found, albeit with a similar outcome. Is this simply a cell type difference or is there more to the story than is currently understood? In any case, these studies and others suggest that focusing on METTL3’s methyltransferase activity might be limiting our understanding of its broader role in physiology and disease.

### 3.2. METTL16 Methylation-Independent Functions

METTL16 has an even stronger case for having m6A-independent functions related to its RNA binding. Anecdotally, in the majority of global studies attempting to catalog the RNA-binding proteome, METTL16 has been identified as an RNA-binding protein, while METTL3, METTL14, WTAP, and FTO have not. Interestingly, in addition to METTL16, many of the reader proteins, including the majority of the YTHD and IGFBP families and the demethylase ALKBH5, have also been identified as RNA-binding proteins [101,102,103,104]. A more recent study taking into account the strength of association confirmed these findings and placed METTL16 in the top 15% of known and predicted mRNA-binding proteins [105]. These results most likely indicate that METTL16 is a bona fide mRNA-binding protein and not just an RNA-modifying enzyme. 

This is not without precedence. MAT2a mRNA was one of the first identified METTL16 RNA-binding and methylation targets, and it exemplifies METTL16’s dual role as a methyltransferase as well as an RNA-binding protein. MAT2A is the catalytic subunit of methionine adenosyltransferase responsible for producing SAM, which is the methyl donor substrate for most methylation reactions in the cell. When SAM levels are adequate, METTL16 binds and methylates adenosines in several hairpin loops, located in the MAT2A 3′ UTR [56,106]. Methylation of the hairpin 1 (HP1) impairs splicing of the terminal intron, leading to intron retention and degradation of the mRNA [56,107], while the methylation of hairpins 2–6 targets the mature mRNA for degradation through recognition of the mRNA by an m6A reader [106,108]. However, if SAM levels become limiting, METTL16 binds to the mRNA but is unable to complete the methylation reaction. The METTL16 bound to HP1 then recruits splicing machinery to the last intron, removing it and allowing for the expression of the full-length mature mRNA and proper translation of the MAT2A protein [56,107]. Thus, through the sensing of SAM levels via its catalytic activity, METTL16 is able to maintain adequate SAM levels for all cellular methylation reactions. When cellular SAM levels are adequate, the MAT2A mRNA is targeted for degradation, both in the nucleus (through aberrant splicing and subsequent degradation) and the cytoplasm (through increased degradation), via METTL16 methylation activity [106]. Conversely, when cellular SAM levels decrease and MAT2A is needed, increased splicing, facilitated by METTL16’s RNA-binding ability, along with an increased mRNA half-life (due to decreased m6A methylation), ensure that the cell can increase its SAM synthesis to meet demands.

Similar to METTL3, METTL16 is thought to methylate its target RNAs in the nucleus; however, we and others have also observed METTL16 in the cytoplasm of a number of different cell types [58,63,109]. Further investigation has revealed that, like METTL3, METTL16 also plays a role in promoting translation in an m6A-independent manner in cancer cells. In hepatocellular carcinoma, METTL16 was found to directly interact with eIF3a/b through its methyltransferase domain (79–289), facilitating the assembly of the translation initiation complex and promoting the translation of specific mRNAs (Figure 2C) [63]. METTL16 binding was localized to the 5′ cap region of targeted mRNAs and tethering either wild-type or catalytically inactive METTL16 to the 5′ UTR of reporter mRNAs significantly enhanced their translation efficiency. Again, this m6A-independent effect of METTL16 was found to be critical for the tumorigenesis of hepatocellular cancer [63].

METTL16 was found to also play a similar role in lung tumorigenesis, where METTL16 depletion attenuated protein synthesis, but that effect could be rescued by either wild-type or catalytically inactive METTL16, again suggesting that the methyltransferase activity is not required [109]. Interestingly, the interactor of METTL16 in these cells was found to be eIF4E2, which represses translation by acting as a competitor of eIF4E. Thus, instead of interacting with the mRNA, the interaction of METTL16 with eIF4E2 impedes the recruitment of eIF4E2 to the cap structure, promoting cap recognition by eIF4E and selective protein synthesis (Figure 2D). In pull-down studies, interactions were also found with a number of other eIFs, including 4E2, 2B4, 2A, 2B5, and 5B, suggesting that there may be additional translation initiation roles for METTL16 as well. Again, the depletion of METTL16 functionally suppressed lung tumorigenesis by downregulating the translation of key oncogenes [109].

Before the discovery of METTL16’s role in translation, it was considered only to be an m6A RNA methyltransferase and only had a few verified targets. However, multiple studies using a variety of techniques have identified a multitude of mRNA binding targets, some of which have never been shown to contain an m6A modification [55,57,58]. Furthermore, correlations among the datasets from these studies and RNA expression changes with METTL16 knockdown/knockout show very little overlap, even when conducted with the same cell types. Thus, there is a great disconnect, either between METTL16’s role in different cell types or, more likely, between METTL16’s methylation activity and its RNA binding activity, and this leads to uncertainty regarding each of their roles in regulating mRNA expression. A recent study investigating a novel aminothiazolone METTL16 RNA-binding activity inhibitor was indeed able to show the separation of METTL16’s RNA binding and methylation activities [110]. Thus, it is clear that more in-depth studies carefully correlating METTL16’s methylation and RNA binding targets are needed, along with studies separating these two roles, before METTL16’s biological role(s) can truly be appreciated. 

Since the discovery of its role in translation, the focus on METTL16 has shifted to its involvement in protein synthesis, but the requirements of the RNA to be bound and recruited by METTL16 have not been thoroughly investigated. It is likely that both RNA-binding domains restrict the RNAs to which METTL16 can bind, but their individual involvement in methylation-dependent and -independent binding has not been investigated. From X-ray crystallography, it has been shown that the N-terminal RNA-binding domain forms a groove that can accommodate a double-stranded RNA form, most likely in a hairpin since the groove is not open-ended [94]. From both homology and crystallography, it has been determined that the C-terminal RNA-binding domain forms a clamp-like structure that is also predicted to bind double-stranded RNA [96]. While most RNAs adopt a secondary structure with stems and hairpins, it is unlikely (but not impossible) that METTL16 binds most, if not all, RNAs to some extent. Therefore, while the association with translation is exciting, continued studies into the specific RNAs being bound (or the requirements set by METTL16’s structure for RNA binding) are crucial.

We, and others, have identified mRNAs that do and do not show significant binding to METTL16, which not only implicates METTL16’s role in the mRNAs’ associated pathways but also contributes to the evidence needed to understand METTL16’s RNA binding. Our work with mutated METTL16 proteins suggests that mutations in different regions of the protein alter the RNA targets bound, suggesting different roles for each domain [111]. Most interesting was the deletion of the VCR linker region, which resulted in the mutant METTL16 protein binding almost every RNA, proving significantly better than the wild-type transgenic METTL16. This gain of function was surprising because the disordered linker region that was deleted is likely necessary for the dynamic motion of RNA binding and unbinding. It seems that rather than prohibiting RNA binding, we prohibited dynamic motion that would have otherwise restricted close contact with the RNA. It is important to note that better binding was observed even to RNAs that do not seem to be METTL16 targets. Thus, it is possible that the linker region, and the flexibility it provides, may be important for restricting RNA binding to a specific subset of targets. 

Similar to METTL3, there are many questions remaining regarding METTL16’s RNA-binding ability and potential methylation-independent roles. Given that METTL16 has two distinct RNA-binding domains, it is possible that, in certain cases, they function independently of each other to determine METTL16 targets. More defined mutations and a closer examination of the effect on RNA binding may shed light on the exact role of each domain and help explain some of the discrepancies found in the literature. There has also been very little work into the dynamics of METTL16’s binding and methylation activity and how they are affected by cell type, cellular signaling, etc. It is quite possible that METTL16’s (and METTL3’s) binding activities may be modulated differently, depending on cellular conditions to fine-tune the protein output. A better understanding of both upstream signaling events and downstream consequences is needed before we can truly understand METTL16’s unique biological role.

## 4. Conclusions

While METTL3 and METTL16 have been the focus of intense study for the past 10 years, much remains to be understood about these intriguing proteins. Both clearly have the ability to bind target RNAs and catalytically transfer a methyl group onto them, thus affecting their regulation, fate, and, ultimately, gene expression of the cell. In addition to binding RNA to m6A modify it, both have also been shown to bind target RNAs that are not methylated but may be regulated in other ways. METTL16 is particularly intriguing in this aspect as it is often identified in global RNA binding screens and has multiple RNA-binding domains that appear to have separate functions. Future studies will need to consider RNA binding in addition to m6A methylation for both METTL3 and METTL16 when evaluating their functions within cells. In addition, focused studies into the functions of METTL3 and METTL16’s distinct RNA-binding domains may help shed light on the role of each domain in determining the target RNAs that are bound and their ultimate fate. 

One area where the RNA-binding ability of METTL3 and METTL16 has clearly been shown to function independently of methylation is in the regulation of translation in cancer cell models (Figure 2). Both METTL3 and METTL16 have been shown to recruit bound (but not necessarily methylated) mRNA to the translation initiation complex to increase the production of proteins necessary for cancer cell proliferation, growth, and migration [53,63,99,100,109]. Interestingly, the studies to date have identified four different mechanisms by which this can occur. Future work will need to expand these studies to other cancer cell types to determine if these mechanisms are universal or restricted to particular scenarios. In addition, as the binding and recruitment of the mRNA appears to be directed toward a subset of targets, it is imperative to understand what determines binding specificity, as it is likely different from that required for the enzyme’s methylation targets.

One big question that remains is what controls the shift of these normally nuclear functioning proteins to the cytoplasm and activation of the RNA binding/translation initiation role. Clearly, METTL3 needs to leave the complex containing METTL14 and WTAP and translocate to the cytoplasm to interact with the initiation machinery. How is this being regulated? Is it simply a stoichiometric effect in which METTL3 levels are much greater than the other components of the complex? Or is it the result of a signaling event? Growth factor and other signaling pathways are often increased in cancer cells and it is possible that METTL3 and/or METTL16 may be downstream targets of those pathways. It could also be the activation of other proteins that associate with the methyltransferases directing them to the cytoplasm and target mRNAs. Future work into understanding how nuclear/cytoplasmic localization is regulated will help shed light on how these enzymes are able to switch their cellular functions and whether this phenomenon is restricted to cancer cells or plays a role in normal cell function as well.

Since their discovery, the cellular m6A methyltransferases METTL3 and METTL16 have been the focus of intense research, and much has been uncovered about their structure, catalytic activity, and cellular function. However, many questions about these enigmatic proteins still remain, especially with regard to their RNA-binding and methylation-independent functions. As future work continues on METTL3 and METTL16, greater care and attention must be paid to these emerging functions so that we may more fully understand their diverse cellular roles.
